# Biomarker Potential of the Soluble Receptor for Advanced Glycation End Products to Predict Bronchopulmonary Dysplasia in Premature Newborns

**DOI:** 10.3389/fped.2021.649526

**Published:** 2021-04-29

**Authors:** Hayato Go, Hitoshi Ohto, Kenneth E. Nollet, Kenichi Sato, Kyohei Miyazaki, Hajime Maeda, Hirotaka Ichikawa, Mina Chishiki, Nozomi Kashiwabara, Yohei Kume, Kei Ogasawara, Maki Sato, Mitsuaki Hosoya

**Affiliations:** ^1^Department of Pediatrics, Fukushima Medical University School of Medicine, Fukushima, Japan; ^2^Fukushima Medical University, Fukushima, Japan; ^3^Department of Blood Transfusion and Transplantation Immunology, Fukushima Medical University School of Medicine, Fukushima, Japan

**Keywords:** rage, premature infants, bronchopulmonary dysplasia, biomarker, serum

## Abstract

Bronchopulmonary dysplasia (BPD) is a common cause of pulmonary disease in preterm infants. The soluble receptor for advanced glycation end products (sRAGE) is implicated in the development of various pulmonary diseases. The objectives of the current study were to investigate perinatal factors associated with serum sRAGE levels at birth and to establish whether serum sRAGE could be a biomarker for BPD. This retrospective single-center study was conducted at Fukushima Medical University Hospital's Department of Pediatrics Neonatal Intensive Care Unit from April 2014 to September 2020. Mechanically ventilated or oxygenated neonates born at <32 weeks gestational age and healthy control neonates were included in this study. Serum sRAGE levels in cord blood were measured using an enzyme-linked immunosorbent assay. Eighty-four preterm infants born at <32 weeks and 40 healthy infants were identified. The 84 born at <32 weeks were categorized as BPD (*n* = 34) or non-BPD (*n* = 50) neonates. The median gestational age (GA) and birthweight (BW) were significantly lower in BPD vs. non-BPD neonates (24.4 vs. 27.6 weeks, *P* < 0.001, 634 vs. 952 g, *P* < 0.001, respectively). Serum sRAGE at birth in all 124 preterm and term infants significantly correlated with BW (*r* = 0.417, *P* < 0.0001) and GA (*r* = 0.415, *P* < 0.0001). Among those born at <32 weeks, median serum sRAGE levels at birth were significantly lower in infants with BPD than without (1,726 vs. 2,797 pg/mL, *P* = 0.0005). Receiver operating characteristic analysis for sRAGE levels at birth in infants with and without BPD revealed that the area under the curve was 0.724 (95% confidence interval 0.714–0.834, *P* = 0.001). However, serum RAGE levels were not associated with severity of BPD. Serum sRAGE levels at birth were significantly correlated with BW and GA. Furthermore, serum sRAGE levels at birth could serve as a biomarker for predicting BPD, but not its severity.

## Introduction

Bronchopulmonary dysplasia (BPD) is a common cause of pulmonary disease in preterm infants, with long-term respiratory and neurodevelopmental consequences (1, 2). BPD has a complex and multifactorial etiology involving maternal inflammation, surfactant deficiency, mechanical ventilation, and oxygen toxicity (3, 4). Premature infants are often exposed to positive pressure ventilation and/or supplemental oxygen, contributing to the development of BPD. A key pathophysiological feature of infants affected with BPD is characterized by perturbations to lung structure including reduced alveolar number, thickened septa, and malformed pulmonary circulation (4). Such structural alterations are accompanied by characteristic inflammatory changes and extensive remodeling of the extracellular matrix (ECM) (4).

The receptor for advanced glycation end-products (RAGE) is a cell surface protein that has been implicated in various conditions, including atherosclerosis, diabetic nephropathy, and pulmonary disease (5–7). RAGE is highly expressed in lung epithelial cells and helps alveolar epithelial cells to maintain their morphology and specific architecture (8). RAGE ligand binding promotes nuclear factor kB-mediated inflammation and generation of reactive oxygen species. Soluble RAGE (sRAGE), without transmembrane or cytoplasmic domains, can act as a decoy receptor for RAGE ligands, thereby attenuating RAGE-mediated inflammation (9). The human RAGE gene (AGER) produces membrane RAGE (mRAGE) with extracellular, transmembrane, and cytosolic domains. Alternative splicing of the AGER gene leads to formation of endogenous soluble RAGE (esRAGE) (9). In adults with chronic obstructive pulmonary disease (COPD), sRAGE is considered as a biomarker for emphysema. Total sRAGE is reduced in the lungs of COPD and idiopathic pulmonary fibrosis patients (10, 11). Further, sRAGE is known to play an important role in lung injury and tissue remodeling (12, 13). However, no mechanisms have been clearly established. In neonates, sRAGE levels were significantly reduced in premature infants with maternal inflammation (14). Furthermore, Benjamin et al. reported that total sRAGE levels were reduced in the airways of preterm infants at risk for developing BPD (15). However, there are no reports investigating the relationships between sRAGE and BPD in premature infants. Furthermore, there are no reports correlating perinatal factors with serum sRAGE at birth in any large cohort.

We hypothesized that serum sRAGE at birth could serve as biomarker for BPD. The objectives of the present study were to correlate perinatal factors with serum sRAGE levels at birth in preterm and healthy infants and to evaluate whether serum sRAGE at birth could be a biomarker for BPD.

## Materials and Methods

### Study Design, Ethics Approval, and Population

This retrospective single-center study was conducted at Fukushima Medical University Hospital's Department of Pediatrics Neonatal Intensive Care Unit (NICU) from April 2014 to September 2020. This research was approved by the Institutional Review Board of Fukushima Medical University, which is guided by local policy, national law, and the World Medical Association Declaration of Helsinki. As our human subjects were neonates, informed consent was solicited from parents or other legal guardians, and documented in writing.

We included infants born at <32 weeks gestational age receiving mechanical ventilation or oxygen therapy. Cord venous blood samples were obtained from these premature neonates and from healthy neonates born from 36.6 weeks to term in our hospital if informed consent was obtained from parents and/or legal guardians and documented in writing. Exclusion criteria were congenital anomalies or neonates who died prior to day of life (DOL) 28. Data for analysis included gestational age (GA), phenotypic sex, body weight at birth (BW), invasive mechanical ventilation at DOL 28, supplemental oxygen at DOL 28, respiratory distress syndrome (RDS), being small for gestational age (SGA), Apgar scores, and various maternal complications including chorioamnionitis (CAM), premature rupture of membranes (PROM), and hypertensive disorders of pregnancy (HDP). CAM was defined by histological diagnosis. BPD was defined in accordance with the National Institutes of Health consensus definition for infants (16). Infants were classified into the following groups at 36 weeks postmenstrual age (PMA): mild BPD was defined as the need for supplemental oxygen at ≥28 days but not at 36 weeks PMA; moderate BPD was defined as the need for supplemental oxygen at 28 days, in addition to supplemental oxygen with an FiO_2_ (fraction of inspired oxygen) ≤ 0.30 at 36 weeks PMA; severe BPD was defined by also needing supplemental oxygen at 36 weeks PMA and needing mechanical ventilation and/or FiO_2_ > 0.30 (16). SGA was defined as a birth weight below −1.5 standard deviations of the mean, corrected for the gestational age and sex in accordance with previously published criteria (17).

### Serum sRAGE Measurements

Using serum samples of cord venous blood at birth stored at −80°C until assay, serum sRAGE levels were measured using an enzyme-linked immunosorbent assay (ELISA; Quantikine; R&D systems, USA) according to manufacturer's protocol. Specifically, a monoclonal antibody raised against the extracellular domain of human RAGE was used to capture sRAGE from serum. Captured sRAGE was detected with a polycloncal antihuman sRAGE antibody. After washing, plates were incubated with streptavidin-HRP, developed with appropriate substrate, and OD450 was determined using an ELISA plate reader (18).

### Statistical Analysis

All data are presented as medians. The Mann–Whitney *U*-test was used to compare continuous variables, and the χ^2^ test was used for nominal variables. The Kolmogorov-Smirnov test was used to judge the normality tests of distribution of serum sRAGE levels at birth-frequency. The Kruskal–Wallis test was used for comparing intergroup variances among non-BPD and BPD subdivided as mild, moderate, and severe. Correlations between GA, BW, and serum sRAGE levels were evaluated using Spearman's correlation coefficient. Multivariate analysis was used to determine perinatal factors significantly associated with serum sRAGE levels at birth. Furthermore, we also performed multivariate analyses to determine factors significantly associated with BPD as potential confounding factors such as BW, GA, invasive mechanical ventilation at DOL 28, Apgar score at 1 min <3, oxygen supplementation at DOL 28, and serum sRAGE levels at birth in premature infants born at <32 weeks, adjusted for mechanical ventilation using stepwise regression. Receiver operating characteristic (ROC) analysis and its area under the curve (AUC) were applied to quantify independent risks for BPD. Data analysis was performed with SPSS (version 21.0) and GraphPad Prism version 8 software.

*P* < 0.05 were considered to be statistically significant.

## Results

**Figure 1:**
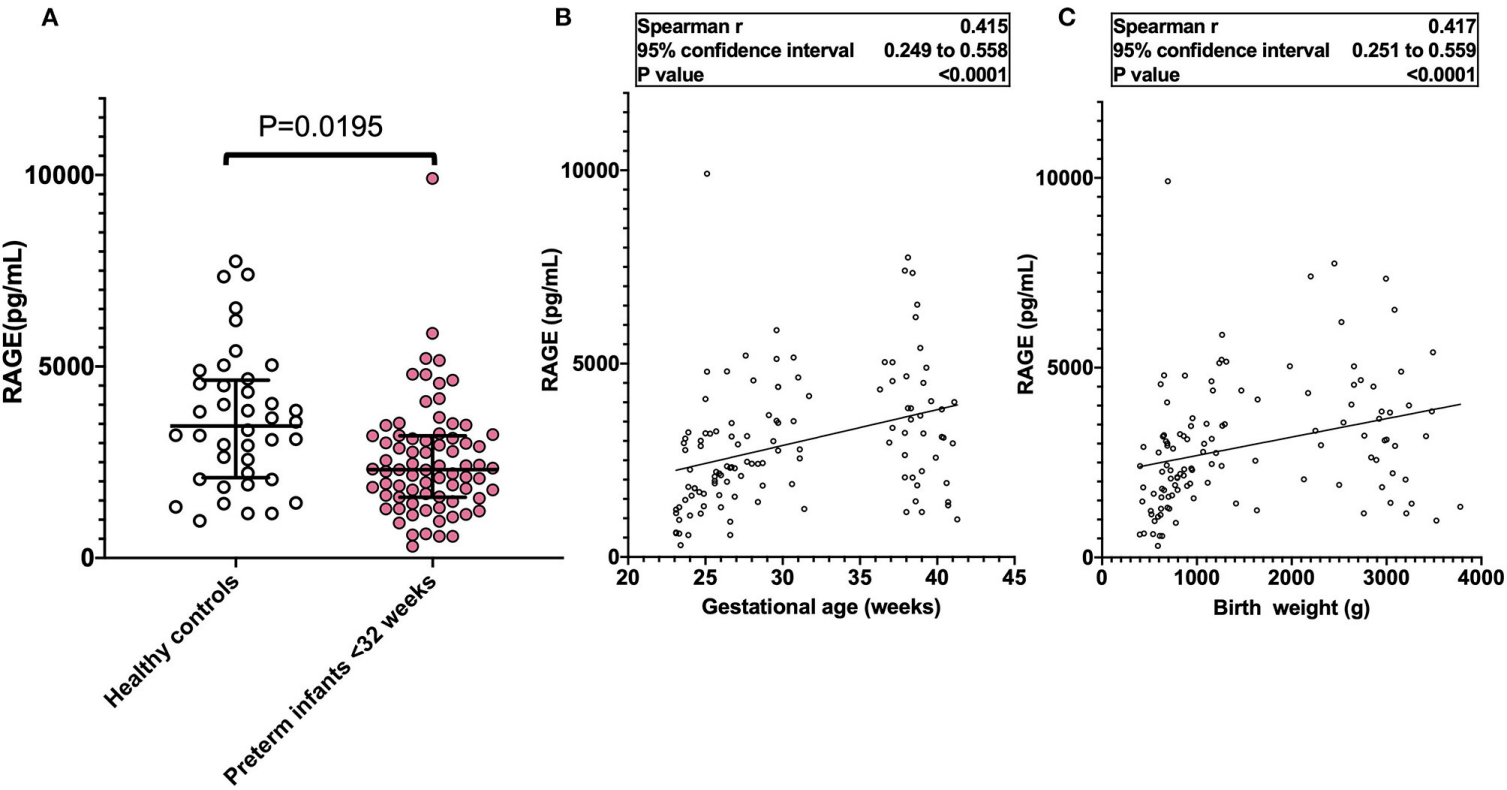
**(A)** Serum sRAGE levels in term (*n* = 40) and preterm infants (*n* = 84, born at <32 weeks). Horizontal bars denote the median and interquartile values in each group of infants. **(B,C)** Serum sRAGE levels at birth were significantly correlated with gestational age and birth weight. The linear relationship between variables was analyzed by Spearman's rank-order correlation.

Of 984 neonates admitted to our NICU between April 2004 and September 2020, our study included 84 preterm infants born at <32 weeks and 40 healthy infants, from whom cord blood was obtained. Table 1 shows their clinical characteristics. Of 84 infants born at <32 weeks, 34 infants developed BPD and 50 infants did not. The median GA and BW in BPD infants were significantly lower than that in non-BPD neonates (24.4 vs. 27.6 weeks, *P* < 0.001, 634 vs. 952 g, *P* < 0.001, respectively). Apgar score at 1 min <3 occurred in BPD infants more often than non-BPD infants (35.0 vs. 8%, *P* < 0.002). Oxygen supplementation and invasive mechanical ventilation at DOL 28 were more likely in BPD infants than in non-BPD infants. There were no significant differences between control and BPD infants by sex, antenatal steroid usage, SGA, PDA, CAM, HDP, or PROM (Table 1). As shown in Figure 1A, the median serum sRAGE levels at birth among preterm infants born at <32 weeks was significantly lower than those among healthy infants (2,377 vs. 2,935 pg/mL, *P* = 0.0195). Levels of sRAGE at birth in 124 preterm and term infants were significantly correlated with BW (*r* = 0.417, *P* < 0.0001) and GA (*r* = 0.415, *P* < 0.0001) (Figures 1B,C). As shown in Table 2, we investigated perinatal factors affecting serum RAGE levels. Multivariate analysis showed that GA and BW were significantly correlated with serum sRAGE levels at birth.

**Table 1:** Characteristics of subjects.

	**Healthy controls (*N* = 40)**	**Non-BPD (*N* = 50)**	**BPD (*N* = 34)**	***P*-value**
Gestational age, median, weeks	38.7	27.6	24.4	**<0.001**
Birth weight, median, g	2,948	952	634	**<0.001**
Male sex, n (%)	18 (45)	24 (48)	17 (50)	0.857
CAM, n (%)	0 (0)	22 (44)	17 (50)	0.647
Antenatal steroids, n (%)	0 (0)	44 (88)	32 (94)	0.857
PROM, n (%)	0 (0)	14 (28)	10 (3)	0.888
HDP, n (%)	0 (0)	8 (16)	1 (3)	0.058
RDS, n (%)	0 (0)	34 (68)	27 (79)	0.250
SGA, n (%)	0 (0)	7 (14)	5 (15)	0.928
PDA, n (%)	NA	31 (62)	16 (47)	0.176
Oxygen supplementation at DOL 28, n (%)	NA	18 (36)	28 (82)	**<0.001**
Invasive mechanical ventilation at DOL 28, n (%)	NA	20 (40)	33 (97)	**<0.001**
Apgar score at 1 min <3, n (%)	0 (0)	4 (8)	12 (35)	**0.002**
Apgar score at 5 min <3, n (%)	0 (0)	3 (6)	5 (15)	0.182

*NA, not applicable; CAM, chorioamnionitis; PROM, premature rupture of membranes; HDP, hypertensive disorders of pregnancy; SGA, small for gestational age; RDS, respiratory distress syndrome; PDA, patent ductus arteriosus. P-values are for differences between BPD and non-BPD neonates. P values in bold are those that show statistical significance*.

**Table 2:** Perinatal factors affecting serum RAGE levels in term and preterm neonates.

			**Univariate analysis**	**Multivariate analysis**	
		**sRAGE levels [IQR] (pg/mL)**	***P*-value**	**Beta**	***P*-value**
Gestational age			<0.001	−2.010	**0.047**
Birth weight			<0.001	2.422	**0.017**
Male sex	Yes	2,766 [1,876–3,743]	0.753	0.994	0.323
	No	2,752 [1,604–3,846]			
CAM	Yes	1,299 [1,380–2,968]	<0.001	−0.759	0.449
	No	3,000 [2,087–4,095]			
Antenatal steroids	Yes	2,314 [1,592–3,157]	<0.001	−0.808	0.421
	No	3,424 [2,237–2,425]			
PROM	Yes	1,859 [1,380–2,334]	0.001	−1.540	0.126
	No	3,000 [1,940–4,123]			
HDP	Yes	3,510 [2,711–4,640]	0.223	0.339	0.735
	No	2,634 [1,657–3,661]			
RDS	Yes	2,766 [1,635–3,468]	0.326	1.709	0.090
	No	2,752 [1,848–4,016]			
SGA	Yes	2,417 [1,899–3,289]	0.748	−1.689	0.094
	No	2,824 [1,657–3,847]			
Apgar score at 1 min <3	Yes	1,792 [1,297–2,927]	0.142	−0.134	0.893
	No	2,839 [1,868–4,016]			
Apgar score at 5 min <3	Yes	2,484 [1,750–2,861]	0.422	−0.562	0.575
	No	2,766 [1,776–3,926]			

*IQR, interquartile range; Beta, standardized coefficient; CAM, chorioamnionitis; PROM, premature rupture of membranes; HDP, hypertensive disorders of pregnancy; SGA, small for gestational age; RDS, respiratory distress syndrome. P-values are for differences between preterm and term neonates. P values in bold are those that show statistical significance*.

**Figure 2:**
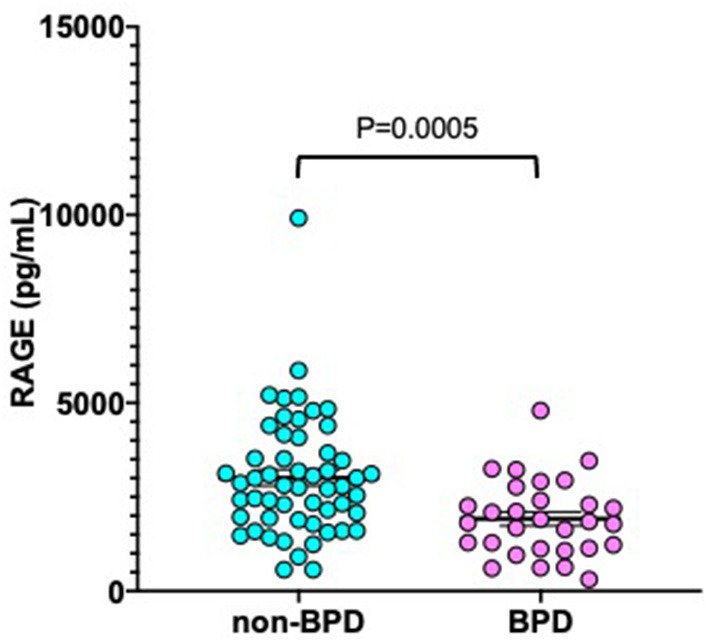
Serum sRAGE levels at birth in non-BPD (*n* = 50) and BPD (*n* = 34) infants born at <32 weeks gestational age. The *P*-values were calculated using the Mann-Whitney *U*-test. BPD, bronchopulmonary dysplasia.

**Figure 3:**
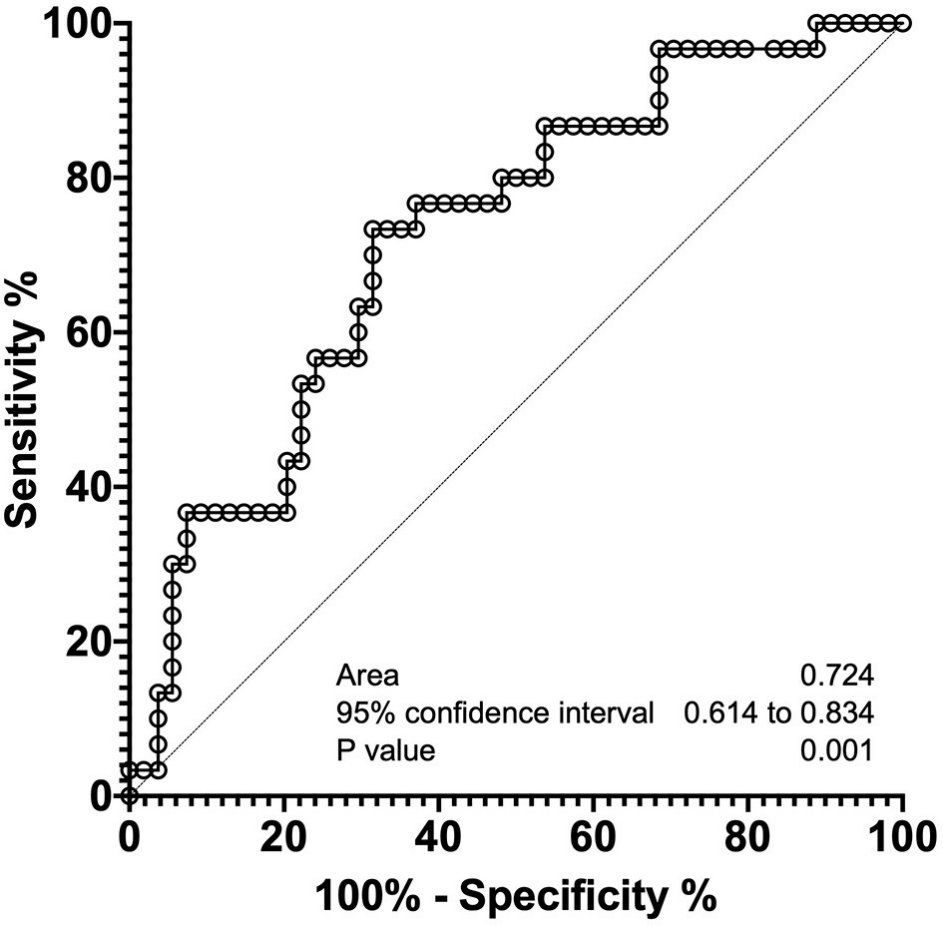
Receiver operating characteristic curve analyses of sRAGE levels at birth, to distinguish infants with and without BPD.

As shown in Figure 2, median sRAGE levels among those with BPD (1,726 pg/mL) were significantly lower than those without BPD (2,797 pg/mL, *P* = 0.0005). Next, we performed multivariate analysis to explore risk factors associated with BPD (Table 3). GA (*P* = 0.014), BW (*P* = 0.015), and serum sRAGE (*P* = 0.028) levels at birth significantly correlated when analyzed using stepwise regression. ROC analysis for sRAGE levels at birth in infants with and without BPD revealed that the AUC was 0.724 (95% confidence interval 0.714–0.834, *P* = 0.001) (Figure 3).

**Table 3:** Risk factors associate with BPD by multivariate stepwise regression.

	**Step 0**	**Step 1**
	***P*-value**	***P*-value**
Gestational age	<0.001	**0.014**
Birth weight	<0.001	**0.015**
Male sex	0.927	0.509
CAM	0.647	0.964
Antenatal steroids	0.486	0.513
PROM	0.773	0.558
HDP	0.054	0.222
RDS	0.227	0.317
SGA	0.957	0.548
PDA	0.202	**0.002**
Oxygen supplementation at DOL28	<0.001	0.372
Invasive mechanical ventilation at DOL28	<0.001	–
Apgar score at 1 min <3	0.002	0.122
Apgar score at 5 min <3	0.193	0.661
Serum RAGE levels at birth (ng/mL)	0.001	**0.028**

*CAM, chorioamnionitis; PROM, premature rupture of membranes; HDP, hypertensive disorders of pregnancy; SGA, small for gestational age; RDS, respiratory distress syndrome; PDA, patent ductus arteriosus. P-value was compared between BPD and non-BPD neonates. Step 0, no variables entered. Step 1, invasive mechanical ventilation entered as a variable. P values in bold are those that show statistical significance*.

**Figure 4:**
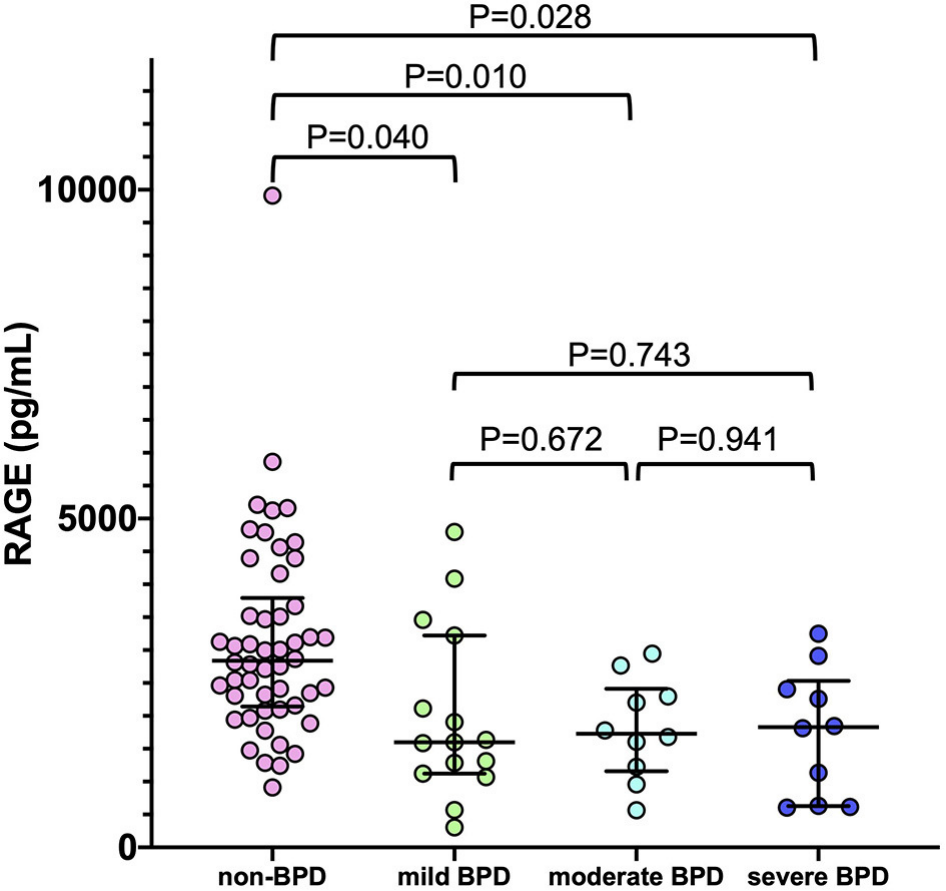
The correlation between serum sRAGE levels at birth and severity of BPD (non-BPD: *n* = 50, mild BPD: *n* = 15, moderate BPD: *n* = 9, severe BPD: *n* = 10). Horizontal bars denote the median and interquartile values in each group of infants. The *P*-values were calculated using the Mann-Whitney *U*-test and Bonferroni correction. BPD, bronchopulmonary dysplasia.

As shown in Figure 4, there was no significant correlation between serum RAGE levels at birth and severity of BPD. Median (IQR) RAGE levels in mild, moderate and severe BPD were 1,599 (1,121–3,221) pg/mL, 1,728 (1,159–2,414) pg/mL, and 1,829 (627.3–2,533) pg/mL, respectively.

## Discussion

To the best of our knowledge, this is the first study to reveal that lower serum sRAGE levels at birth among those born at <32 weeks gestational age is an independent risk factors for BPD. This result is consistent with emerging data implicating lower sRAGE in the pathogenesis of lung disease (19). Benjamin et al. suggested that sRAGE levels in tracheal aspirations were reduced in preterm infants (15). In adult COPD and idiopathic pulmonary disease, serum and lung sRAGE levels were reduced (19). In particular, sRAGE has been implicated in the pathogenesis of lung inflammation and injury leading to tissue remodeling in obstructive or inflammatory airway disease and fibrotic lung disease (19). RAGE is known to have a homeostatic function in the lung by enhancing the adherence of type I epithelial cells to the extracellular matrix (20), and is implicated in the differentiation of type II epithelial cells as critical step in the process of alveolar repair (21, 22). On the other hand, in a murine model, RAGE knockout mice showed less but larger and thicker alveoli, which suggests that RAGE supports alveolarization (23). Previous studies reported that cleaving full-length RAGE (the transmembrane form) is the main way to produce the soluble form from alveolar epithelial cells in healthy human lung (24). This might suggest reduced serum sRAGE in BPD patients could be interpreted as a lack of RAGE synthesis or the loss of alveolar epithelial cells. BPD is characterized by arrested lung alveolarization, which generates lungs that are incompetent for effective gas exchange (25). Although our findings suggest that reduced sRAGE levels might reflect less alveolarization in BPD infants, we could not demonstrate a mechanism to explain why reduced serum sRAGE levels are present in BPD infants.

This also appears to be the first study to correlate serum sRAGE levels at birth with other perinatal factors in neonates. Gestational age and birthweight were positively correlated with serum sRAGE levels. However, previous research suggested that plasma sRAGE levels at birth were negatively correlated with gestational age (26), contrary to our results. This discrepancy may be due to their lower sample size (*n* = 12) of infants born at <30 weeks, whereas our cohort included 74 infants born at <30 weeks. On the other hand, sRAGE levels have been associated with increased inflammation in both the mother and the infant at birth (14). In human preterm neonates exposed to maternal chorioamnionitis or funisitis, sRAGE levels are lower in cord blood (14). We also found associations between serum CAM, PROM, and sRAGE in univariate analysis, however, no associations emerged in multivariate analysis. Recently, some studies have reported that lower sRAGE was associated with poor outcome in infants with sepsis and cerebral palsy (27, 28).

Egron et al. suggested that serum sRAGE levels in children with bronchitis were negatively correlated with age during the first year of life (29), and that serum sRAGE levels in neonates with bronchitis approached 1,500 pg/mL. However, their study did not include healthy neonatal controls. In contrast, Buhimschi et al. reported that cord blood sRAGE levels in neonates (median GA: 32.0 weeks) without maternal and fetal inflammation were almost 2,200 pg/mL (30). In the present study, median serum sRAGE levels at birth at <32 weeks were 2,377 pg/mL. This result is consistent with Buhimschi's study. Further, they also reported that cord blood sRAGE levels in neonates (median GA: 27.6 weeks) with maternal inflammation were under 1,500 ng/mL (2). Thus, serum sRAGE levels in infants were lower in the context of inflammatory conditions. Specific developmental changes associated with serum sRAGE levels warrant further investigation.

There are several limitations in our study, including its single-center sample size and retrospective nature. Specifically, the number of severe BPD infants was too small for any correlations to emerge akin to those seen in adults with severe pulmonary disease. To generalize our observations, a larger sample size including multiple centers would be needed. We could not find a significant association between serum sRAGE levels and the severity of BPD. In adult pulmonary disease such as chronic obstructive pulmonary disease and idiopathic pulmonary fibrosis, blood sRAGE is significantly decreased (21, 31). Third, we could not investigate any correlation between sRAGE levels and high-mobility group box-1 (HMGB1) levels. RAGE ligands include advanced glycation end products (AGEs), HMGB1, and S100 family proteins. Ligation of RAGE elicits activation of immune and inflammatory responses, induction of oxidative stress and tissue remodeling (32). Further, reduced sRAGE is associated with increased levels of HMGB1 in inflammatory responses (32, 33). Moreover, previous research reported that HMGB1 levels in tracheal aspirates from premature infants with BPD were higher than those in without BPD (34). Finally, we could not retrospectively investigate sRAGE levels at 28 days of life or at a corrected 36-week gestational age. This confounds analysis of BPD in the context of a hospital course that might include mechanical ventilation performed solely for apnea, or for sepsis, necrotizing enterocolitis, confounding effects of organ injuries such as acute kidney injury or intracranial hemorrhage, and feeding or nutritional deficits. Further study including samples at other time points, such as 28 days of life or a corrected 36-week gestational age would be better to establish whether sRAGE is associated with various factors related to BPD.

In conclusion, our study suggested that serum sRAGE levels at birth were significantly correlated with birth weight and gestational age and could serve as a biomarker for predicting BPD, however, not as a biomarker for severity of BPD. The mechanism by which serum sRAGE at birth is lower in BPD infants awaits further elucidation.

## Data Availability Statement

The raw data supporting the conclusions of this article will be made available by the authors, without undue reservation.

## Ethics Statement

The studies involving human participants were reviewed and approved by Institutional Review Board of Fukushima Medical University. Written informed consent to participate in this study was provided by the participants' legal guardian/next of kin.

## Author Contributions

HG designed the study, carried out the analyses, drafted the manuscript, and reviewed and revised the manuscript. HO, KN, and MH critically reviewed the initial draft and provided ongoing scientific and editorial guidance. KS, HI, and KM carried out the analyses and reviewed the manuscript. HM, KO, MS, and YK collected the samples and reviewed the manuscript. All authors approved the final manuscript as submitted and agree to accountable for all aspects of the work.

## Conflict of Interest

The authors declare that the research was conducted in the absence of any commercial or financial relationships that could be construed as a potential conflict of interest.
